# Diagnostic accuracy of ultrasonography compared with nerve conduction studies in carpal tunnel syndrome

**DOI:** 10.3389/fneur.2026.1831290

**Published:** 2026-05-08

**Authors:** Aysu Çetiner Şeker, Ayşe Oytun Bayrak

**Affiliations:** 1 Department of Neurology, Samsun Education and Research Hospital, Samsun, Türkiye; 2 Department of Neurology, Faculty of Medicine, Ondokuz Mayıs University, Samsun, Türkiye

**Keywords:** carpal tunnel syndrome, hypothenar muscle thickness, median nerve cross-sectional area, thenar muscle thickness, ultrasonography

## Abstract

**Introduction:**

Carpal tunnel syndrome (CTS) is the most common entrapment neuropathy of the upper extremity. Ultrasonography (USG) enables measurement of the median nerve cross-sectional area (CSA) and quantitative assessment of hand muscles. This study aimed to evaluate the diagnostic accuracy of USG findings compared with electrophysiological studies in CTS.

**Methods:**

In this prospective cross-sectional study, we included 136 wrists from 76 patients presenting to the Neurology Department of Ondokuz Mayıs University between July 2023 and December 2023. Ultrasonographic findings were compared with electrophysiological results.

**Results:**

The cut-off value for median nerve CSA in the diagnosis of CTS was 0.0905 cm^2^, with a sensitivity of 90.3% and specificity of 90.1%. ROC curve analysis revealed an area under the curve (AUC) of 0.948 (95% CI: 0.924–0.973), suggesting excellent discriminative ability. Median nerve CSA was significantly higher in the patient group compared to controls. Thenar muscle thickness was significantly reduced in the CTS group, particularly in severe and extreme stages. No significant difference was observed in hypothenar muscle thickness between groups.

**Conclusion:**

Ultrasonography showed excellent diagnostic accuracy in detecting carpal tunnel syndrome, with high agreement with electrophysiological findings. Among the evaluated parameters, median nerve cross-sectional area and thenar muscle thickness were strong diagnostic indicators. These results support the role of ultrasonography as a reliable, non-invasive diagnostic tool in CTS. Further large-scale, multicenter studies are warranted to confirm these findings and to define standardized diagnostic thresholds.

## 1 Introduction

Carpal tunnel syndrome (CTS) is the most common entrapment neuropathy of the upper extremity (1). It is a syndrome that develops as a result of the median nerve being compressed in the canal formed by the tendons of the flexor muscles, the carpal bones, and the transverse carpal ligament in the wrist, where it passes under the flexor retinaculum (2). CTS is more common in women than in men; it is mostly bilateral, and it is frequently seen in middle-aged women (3–5). Many conditions can cause CTS, but most cases are idiopathic (5).

Electroneuromyography (ENMG) is the gold standard method for diagnosing CTS. However, studies have reported a false negative rate of 10–25% in ENMG studies (5). Imaging methods can also be used in the diagnosis of CTS; specifically, ultrasonography (USG) and magnetic resonance imaging can be employed. However, magnetic resonance imaging cannot be routinely applied in the diagnosis of CTS, because it is expensive and not easily accessible (6). In contrast, USG is a comfortable and non-invasive test for patients. Thus, USG has become an important tool in the diagnosis of CTS because it is easily accessible and painless, as well as because it has a short application time. It also has an important role in the investigation of the secondary causes of CTS diagnosis and the detection of anatomical variations of nerves, and it can guide interventional procedures (7). Despite the widespread use of electroneuromyography (ENMG) as the reference standard, it has certain limitations, including false-negative results and patient discomfort. Although ultrasonography (USG) has emerged as a non-invasive and accessible alternative, its diagnostic accuracy and the optimal measurement parameters for CTS remain to be clearly defined.

Recent studies have further supported the diagnostic value of ultrasonography in CTS, demonstrating strong correlations between median nerve cross-sectional area and electrophysiological parameters, as well as high diagnostic accuracy in ROC-based analyses (8). In addition, emerging techniques such as shear wave elastography and multiparametric ultrasound have been shown to improve early detection and severity assessment of CTS (9).

Therefore, the aim of this study was to evaluate the diagnostic accuracy of ultrasonographic parameters, including median nerve cross-sectional area (CSA) and thenar muscle thickness, in patients with carpal tunnel syndrome and to compare these findings with electrophysiological results. We hypothesized that these ultrasonographic parameters would demonstrate high diagnostic accuracy and could serve as reliable adjunctive tools in the clinical diagnosis of CTS.

## 2 Materials and methods

This study was conducted in accordance with the STROBE guidelines for observational studies.

### 2.1 Study design and setting

This prospective cross-sectional study was conducted at the Neurology Department of Ondokuz Mayıs University between July 2023 and December 2023.

### 2.2 Participants

The study population consisted of patients with carpal tunnel syndrome (CTS) and healthy controls. For the patient group, individuals with a clinical suspicion of CTS based on history and physical examination who subsequently underwent electrophysiological evaluation to confirm the diagnosis were included. Eligible participants were adults (≥18 years) with a confirmed clinical and electrophysiological diagnosis of CTS. No upper age limit was applied.

Patients were excluded if they had a history of wrist surgery, trauma, or fracture, as well as any condition that could affect the peripheral nervous system, such as hypothyroidism, diabetes mellitus, chronic renal failure, or polyneuropathy. Pregnant women were also excluded due to the potential influence of pregnancy-related physiological changes on the development of carpal tunnel syndrome.

The control group comprised age- and sex-matched healthy volunteers with no history of peripheral nervous system disease, no upper extremity neuropathic symptoms, and normal neurological examination findings.

**Figure 1:**
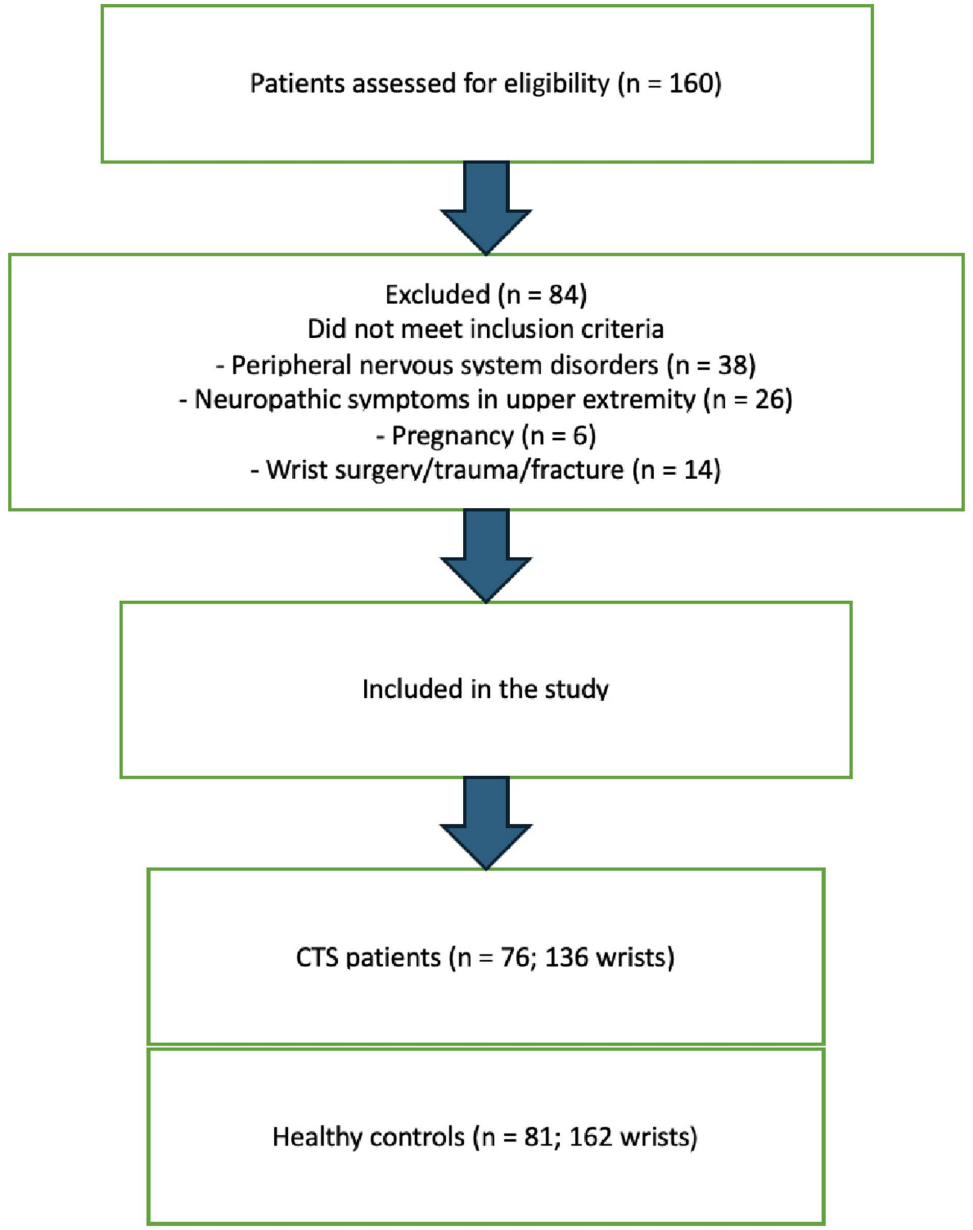
Flow diagram of participant recruitment, exclusion, and final inclusion in the study.

A total of 160 patients were initially assessed for eligibility. Of these, 84 were excluded based on the predefined criteria. Finally, 76 patients with CTS (136 wrists) and 81 healthy controls (162 wrists) were included in the study. The participant selection and exclusion process is summarized in a flow diagram (Figure 1).

The unit of analysis was the wrist. In patients with bilateral CTS, both wrists were included as separate observations.

### 2.3 Ethics statement

This study was approved by the Ondokuz Mayıs University Local Ethics Committee (approval no. OMÜ/KAEK 2023/80). Written informed consent was obtained from all participants, and the study was conducted in accordance with the principles of the Declaration of Helsinki. This research did not receive any specific grant from funding agencies in the public, commercial, or not-for-profit sectors.

### 2.4 Electrophysiological assessment

Electrophysiological examinations were performed in all participants by the same researcher using a Cadwell device (Cadwell Industries, WA, United States) at the Ondokuz Mayıs University electrophysiology laboratory.

During the assessments, the room temperature was maintained at 25 °C, and the palm temperature of the participants was kept at ≥31 °C.

Motor and sensory nerve conduction studies of the median and ulnar nerves were performed. In cases where routine nerve conduction studies were normal, additional comparative tests were conducted.

All examinations were carried out with participants in the supine position, using standard supramaximal percutaneous stimulation with surface electrodes.

Motor and sensory nerve conduction studies of the median and ulnar nerves were performed using standard techniques.

In the median nerve motor conduction study, the active electrode was placed over the abductor pollicis brevis (APB) muscle, with the reference electrode positioned on the first metacarpophalangeal joint. Distal stimulation was applied at the wrist 8 cm proximal to the recording electrode, and proximal stimulation was performed at the antecubital fossa.

In the ulnar nerve motor conduction study, the active electrode was placed over the adductor digiti minimi (ADM) muscle, with the reference electrode on the fifth metacarpophalangeal joint. Stimulation was applied at the wrist and proximally at the elbow with the arm in flexion.

Sensory nerve conduction studies were performed using ring electrodes with the antidromic technique. For the median nerve, recordings were obtained from the third digit, with stimulation at the wrist (13 cm) and palm. For the ulnar nerve, recordings were obtained from the fifth digit, with stimulation at the wrist (11 cm).

The CMAP amplitude was measured from baseline to negative peak, and SNAP amplitude from peak to peak. Motor latency was defined as onset latency, and sensory latency as peak latency.

All electrophysiological parameters were interpreted according to the reference values of our laboratory. Accordingly, in our electrophysiology laboratory, in the median nerve motor study, a CMAP amplitude over 4 mV, a conduction velocity over 49 m/s, and a distal latency under 4.4 ms were considered normal. In the ulnar nerve motor conduction study, a CMAP amplitude over 6 mV, a conduction velocity over 49 m/s, and a distal latency under 3.3 ms were considered normal. In the median nerve sensory conduction study, a SNAP amplitude of over 20 mV obtained by stimulation from the wrist, a conduction velocity of over 50 m/s, and a distal latency of under 3.5 ms were considered normal. The difference between the distal sensory latency of the median nerve at the wrist and palm level was considered to be in favor of CTS if it was over 1.6 (10). In the ulnar nerve sensory conduction study, a SNAP amplitude above 17 mV, a conduction velocity above 50 m/s, and a distal latency below 3.1 m/s were considered normal.

When routine nerve conduction studies were normal, additional comparison tests were performed. For this purpose, the median–ulnar sensory latency comparison test (fourth digit) was applied. The recording electrode was placed over the metacarpophalangeal joint of the fourth digit. SNAPs were obtained by stimulating the median and ulnar nerves at the wrist from equal distances (11–13 cm) proximal to the recording electrode. A latency difference of ≥0.4 ms between the median and ulnar nerves was considered significant for the diagnosis of CTS (5, 11).

The second comparison test involved the assessment of distal motor latencies between the second lumbrical and interosseous muscles. The active electrode was placed slightly lateral and distal to the midpoint of the third metacarpal, while the reference electrode was positioned over the proximal interphalangeal joint of the second digit. Both the median and ulnar nerves were stimulated at the wrist from the same distance (8–10 cm). A latency difference of ≥0.4 ms was considered significant. According to the results of the electrophysiological studies, CTS was grouped into the five following stages (12):

(1) Minimal: Normal routine sensory and motor conduction studies, with CTS detected only by comparison tests.
(2) Mild: Slowed sensory conduction (palm–wrist or finger–wrist segment) with normal distal CMAP latency.
(3) Moderate: Slowed sensory conduction with prolonged distal CMAP latency.
(4) Severe: Absence of SNAP with prolonged distal CMAP latency.
(5) Extreme: Absence of both thenar CMAP and SNAP amplitudes.

For statistical analysis, the severe and extreme stages were combined into a single subgroup.

### 2.5 Ultrasonographic assessment

Ultrasonographic (USG) examinations were performed in all participants by the same researcher using a 7–14 MHz linear probe at the neurophysiology laboratory of the Neurology Department of Ondokuz Mayıs University.

Three parameters were evaluated: median nerve cross-sectional area (CSA), thenar muscle thickness, and hypothenar muscle thickness.

During the examination, the wrist was positioned in a neutral position on a firm surface. Care was taken to avoid excessive probe pressure during measurements. The median nerve was identified in the axial plane just beneath the flexor retinaculum. The median nerve was scanned in the axial plane from the radioulnar joint to the pisiform level and further to the level of the hamate hook.

The cross-sectional area (CSA) of the median nerve was measured at the carpal tunnel inlet, corresponding to the level of the pisiform, where the nerve is typically most enlarged. Measurements were performed using manual (freehand) tracing along the inner border of the hyperechoic epineurium. Ultrasonographic examinations were performed on the same day as the electrophysiological studies.

In ultrasonographic evaluations, the probe was positioned parallel to the fifth metacarpal in the hypothenar region and parallel to the first metacarpal in the thenar region.

Thenar muscle thickness was measured as the distance from the superficial surface of the abductor pollicis brevis (APB) muscle to the underlying metacarpal cortex at the point of maximal thickness.

Similarly, hypothenar muscle thickness was measured from the superficial surface of the adductor digiti minimi (ADM) muscle to the metacarpal cortex at the point of maximal thickness.

### 2.6 Variables and definitions

The main variables evaluated in this study were median nerve cross-sectional area (CSA), thenar muscle thickness, hypothenar muscle thickness, and electrophysiological CTS staging.

Median nerve CSA was defined as the cross-sectional area of the median nerve measured at the level of the pisiform using ultrasonography.

Thenar muscle thickness was defined as the distance from the superficial surface of the abductor pollicis brevis muscle to the underlying metacarpal cortex at the point of maximal thickness.

Hypothenar muscle thickness was defined as the distance from the superficial surface of the adductor digiti minimi muscle to the metacarpal cortex.

The CTS severity was classified into five stages (minimal, mild, moderate, severe, and extreme) based on electrophysiological findings.

### 2.7 Statistical analysis

Statistical analyses were performed using GraphPad Prism version 8 (GraphPad Software, Inc., CA, United States).

Data distribution was assessed using the Kolmogorov–Smirnov test. Continuous variables are presented as mean ± standard deviation or median (interquartile range), as appropriate.

Categorical variables (e.g., gender) were compared using Fisher’s exact test. Differences in age between groups were analyzed using the independent samples *t*-test.

For comparisons among multiple groups, one-way analysis of variance (ANOVA) followed by the Tukey–Kramer *post hoc* test was used for normally distributed data, whereas the Kruskal–Wallis test followed by Dunn’s post hoc test was applied for non-normally distributed data.

Receiver operating characteristic (ROC) curve analysis was performed to evaluate the diagnostic performance of median nerve cross-sectional area, with comparisons made between the CTS and control groups.

Each wrist was analyzed as an independent unit. In patients with bilateral carpal tunnel syndrome, both wrists were included as separate observations. Although this approach may introduce within-subject correlation, it is consistent with previous studies and was considered appropriate for the study design.

The patient group was classified into minimal, mild, moderate, severe, and extreme CTS stages. Receiver operating characteristic (ROC) analysis was performed to determine the optimal cutoff value of median nerve CSA, and sensitivity, specificity, and positive and negative predictive values were calculated. The correlation between CSA and thenar muscle thickness was evaluated using Spearman’s correlation test. Continuous variables are presented as mean ± standard deviation or median (interquartile range), as appropriate. A *p*-value <0.05 was considered statistically significant.

## 3 Results

### 3.1 Demographic characteristics

The study included 76 patients with CTS (136 wrists) and 81 healthy controls (162 wrists). The flow of participants through the study is presented in Figure 1. No significant differences were observed between the groups in terms of age or gender distribution (*p* > 0.05). Demographic characteristics are summarized in Table 1.

**Table 1:** Comparison of electrophysiological parameters among CTS subgroups and control wrists.

Parameter	Normal wrists (*n* = 18)	Minimal CTS (*n* = 42)	Mild CTS (*n* = 32)	Moderate CTS (*n* = 49)	Severe + Extreme CTS (*n* = 13)
Median sensory latency (msn)	2.92 ± 0.42	3.09 ± 0.41	3.93 ± 0.48^a,b^	4.47 ± 0.63^a,b,c^	–
Median sensory amplitude (μV)	37.32 ± 19.14	31.57 ± 13.24	17.38 ± 7.39^a,b^	11.74 ± 5.81^a,b^	–
Median nerve sensory velocity (m/sn)	42.28 ± 7.20	39.68 ± 7.64	33.13 ± 4.29^a,b^	29.38 ± 4.60^a,b^	–
Ulnar sensory latency (msn)	2.42 ± 0.24	2.40 ± 0.36	2.40 ± 0.29	2.47 ± 0.30	2.40 ± 0.34
Ulnar sensory amplitude (μV)	32.79 ± 14.41	31.45 ± 12.48	28.83 ± 10.85	28.00 ± 10.85	27.72 ± 12.27
Ulnar nerve sensory velocity (m/sn)	44.44 ± 3.70	46.24 ± 6.27	45.63 ± 4.61	45.94 ± 5.10	44.92 ± 5.50
Median motor latency (msn)	3.07 ± 0.42	3.45 ± 0.48	3.81 ± 0.45	5.03 ± 0.76^a,b,c^	7.10 ± 2.02* ^a,b,c^
Median motor amplitude (μV)	8.94 ± 2.53	8.20 ± 1.20	7.73 ± 1.68	6.77 ± 1.60^a,b^	4.50 ± 2.91* ^a,b,c^
Median nerve motor velocity (m/sn)	58.39 ± 4.60	54.69 ± 6.38	54.22 ± 5.78	50.66 ± 8.50^a,b^	48.90 ± 6.45* ^a,b^
Ulnar motor latency (msn)	2.14 ± 0.39	2.33 ± 0.39	2.42 ± 0.29	2.42 ± 0.33	2.39 ± 0.49
Ulnar motor amplitude (μV)	10.04 ± 2.22	10.07 ± 1.85	10.03 ± 1.72	10.22 ± 1.89	9.93 ± 1.82
Ulnar nerve motor velocity (m/sn)	62.06 ± 6.96	64.76 ± 6.07	65.94 ± 8.13	62.74 ± 6.85	60.62 ± 4.48

^a^*p* < 0.05 vs. control group; ^b^*p* < 0.05 vs. minimal CTS; ^c^*p* < 0.05 vs. mild CTS.

CTS, Carpal Tunnel Syndrome, mV, millivolt, μV, microvolt, ms, milisecond, m/sn, meter/s.

^*^For patients with severe CTS.

### 3.2 Electrophysiological examination

The distribution of CTS severity was as follows: minimal (*n* = 42), mild (*n* = 32), moderate (*n* = 49), severe (*n* = 11), and extreme (*n* = 2) wrists.

Electrophysiological parameters, including sensory latency, DSAP amplitude, and conduction velocity, showed significant differences between CTS stages and healthy controls (*p* < 0.05). Median nerve distal motor latency and CMAP parameters were significantly impaired in moderate and severe CTS stages compared to controls and milder stages (*p* < 0.05). Detailed electrophysiological findings are presented in Table 1.

### 3.3 Ultrasonographic examination

**Figure 2:**
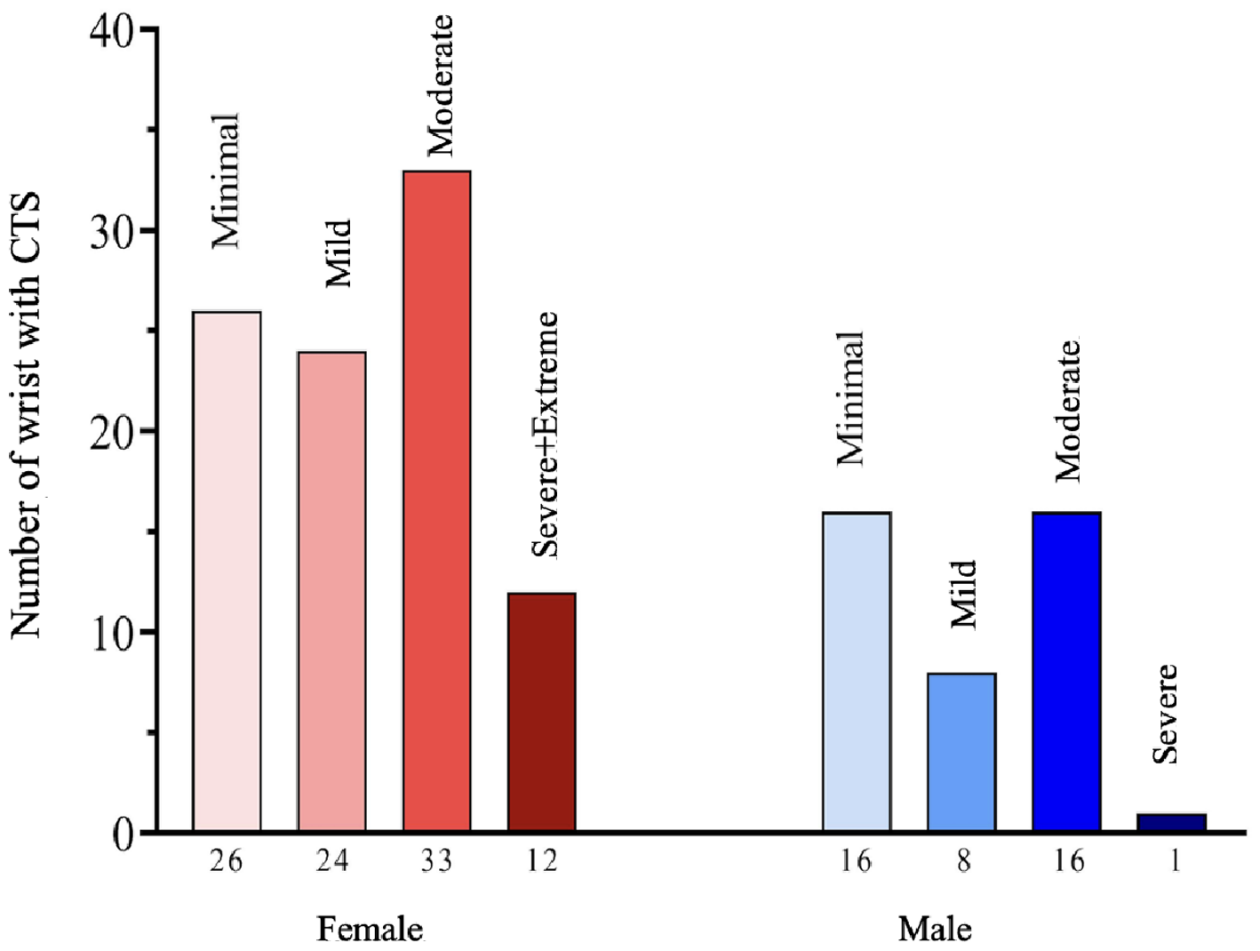
Distribution of carpal tunnel syndrome severity stages according to gender in the patient group.

Median nerve CSA was significantly higher in all CTS subgroups compared to controls (*p* < 0.05), with a progressive increase observed with increasing disease severity. CSA values were significantly greater in severe and extreme CTS stages compared to other subgroups (Figure 2).

Thenar muscle thickness was significantly reduced in all CTS subgroups and showed further reduction in advanced stages (*p* < 0.05). No significant differences were observed in hypothenar muscle thickness between groups.

**Figure 3:**
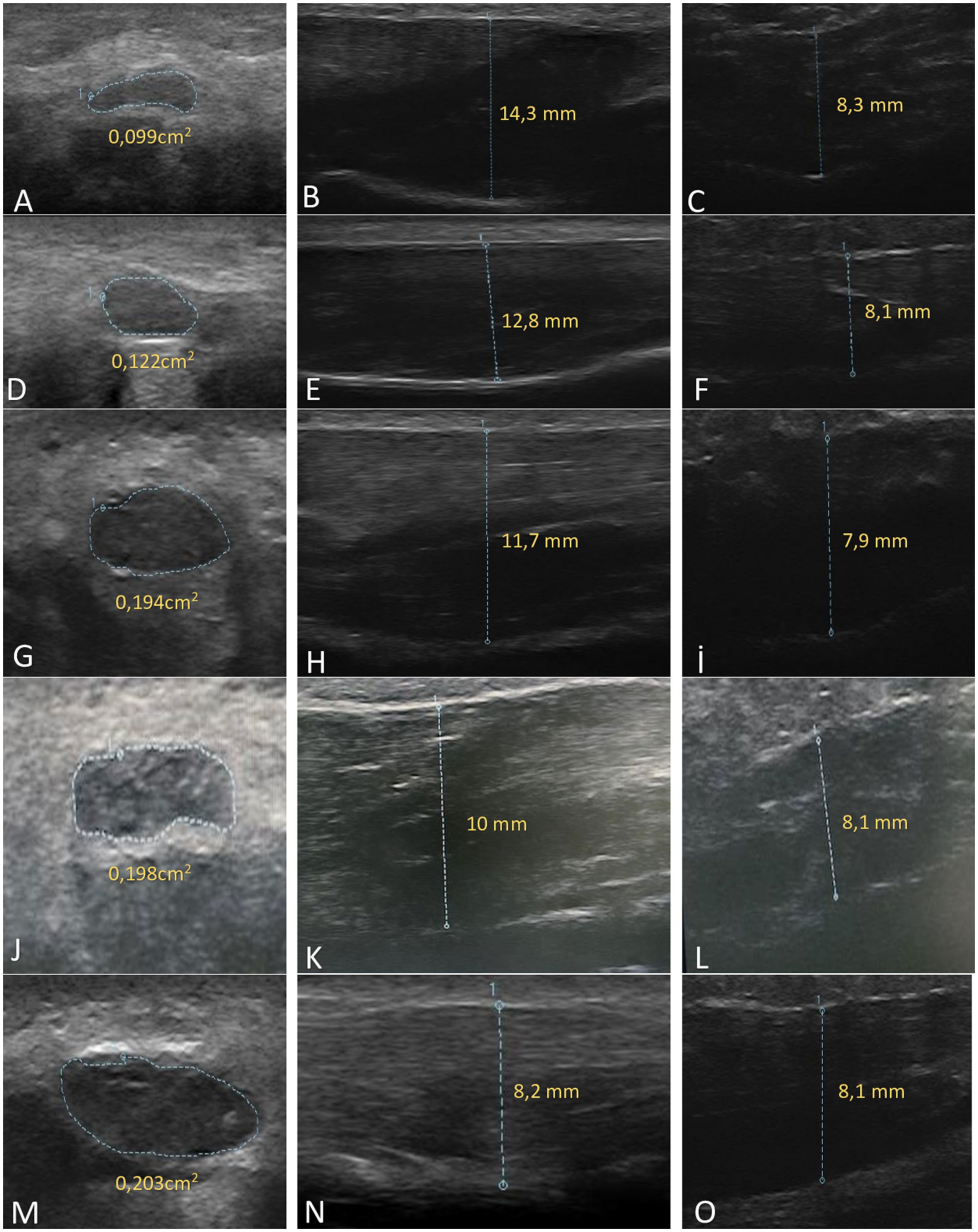
Ultrasonographic images demonstrating median nerve cross-sectional area, thenar muscle thickness, and hypothenar muscle thickness across different stages of carpal tunnel syndrome: minimal stage **(A–C)**, mild stage **(D–F)**, moderate stage **(G–I)**, severe stage **(J–L)**, and extreme stage **(M–O)**.

**Figure 4:**
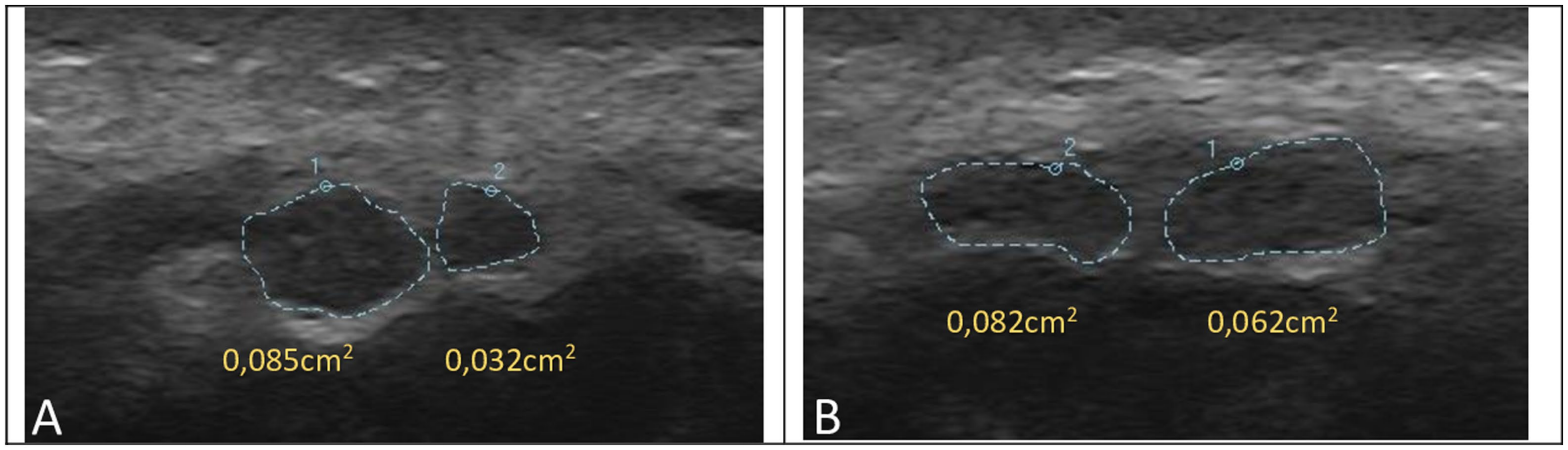
Ultrasonographic examples of bifid median nerve. **(A)** Median nerve cross-sectional area in a patient with mild CTS. **(B)** Median nerve cross-sectional area in a patient with minimal CTS.

A bifid median nerve was identified in 5.1% of CTS wrists and 2.4% of controls. Ultrasonographic findings are summarized in Tables 2, 3 and Figures 3, 4.

**Table 2:** Median nerve cross-sectional area, thenar muscle thickness, and hypothenar muscle thickness across study groups.

	Control (*n* = 162)	Minimal CTS (*n* = 42)	Mild CTS (*n* = 32)	Moderate CTS (*n* = 49)	Severe + Extreme CTS (*n* = 13)
Cross-sectional area of median nerve (cm^2^)	0.073 ± 0.067	0.114 ± 0.043^a^	0.126 ± 0.034^a^	0.136 ± 0.040^a^	0.176 ± 0.052^a,b,c,d^
Thickness of thenar eminence (mm)	13.89 ± 1.60	11.64 ± 3.19^a^	11.36 ± 3.28^a^	12.29 ± 3.28^a^	7.81 ± 1.73^a,b,c,d^
Thickness of hypothenar eminence (mm)	7.04 ± 1.35	6.55 ± 2.15	6.33 ± 2.09	6.58 ± 1.39	6.39 ± 1.27

^a^*p* < 0.05 vs. control group; ^b^*p* < 0.05 vs. minimal CTS; ^c^*p* < 0.05 vs. mild CTS; ^d^*p* < 0.05 vs. moderate CTS.

**Table 3:** Distribution of median nerve cross-sectional area across study groups based on the determined cutoff value.

		ENMG		Total
		CTS	Control	
USG	CTS	121	14	135
	Control	13	148	161
	Total	134	162	

### 3.4 Diagnostic performance and correlation analysis

**Figure 5:**
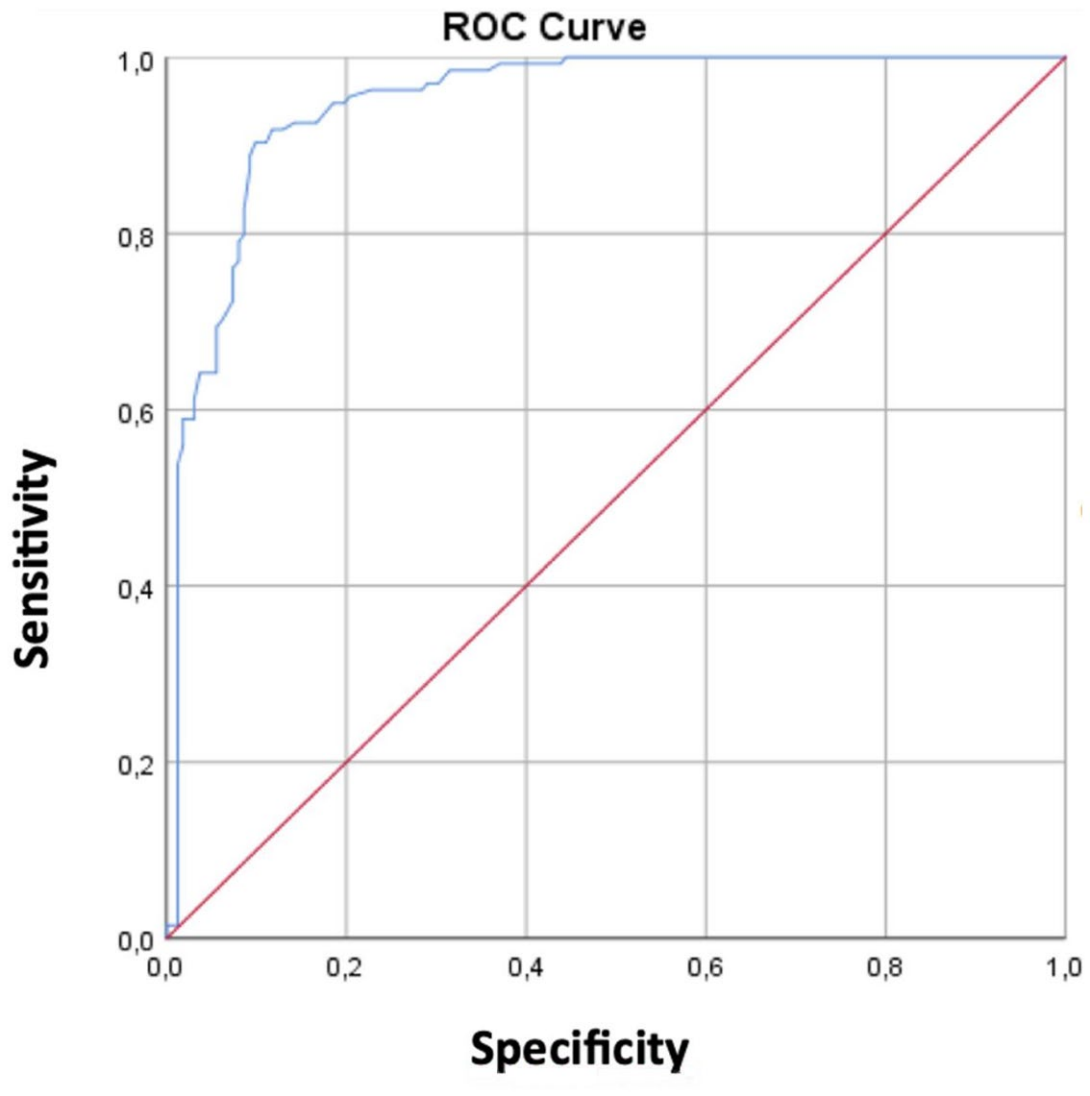
Receiver operating characteristic (ROC) curve for the median nerve cross-sectional area. The analysis demonstrated an AUC of 0.948 (95% CI: 0.924–0.973), indicating excellent diagnostic performance.

ROC curve analysis demonstrated excellent diagnostic performance of median nerve CSA, with an optimal cutoff value of 0.0905 cm^2^, yielding a sensitivity of 90.3% and specificity of 90.1%. The positive and negative predictive values were 91.3 and 90.2%, respectively (Table 3 and Figure 5).

ROC analysis results are summarized in Table 4. The median nerve CSA demonstrated excellent diagnostic performance. Confidence intervals for sensitivity and specificity were additionally calculated and are presented in Table 4.

**Table 4:** ROC analysis of median nerve cross-sectional area for the diagnosis of carpal tunnel syndrome, including AUC, 95% confidence interval, sensitivity, specificity, and predictive values.

	Cut-off (cm^2^)	AUC	95% CI	Sensitivity (%)	Specificity (%)	PPV (%)	NPV (%)	*p*-value
Median nerve CSA	0.0905	0.948	0.924–0.973	90.3	90.1	91.3	90.2	<0.001

**Figure 6:**
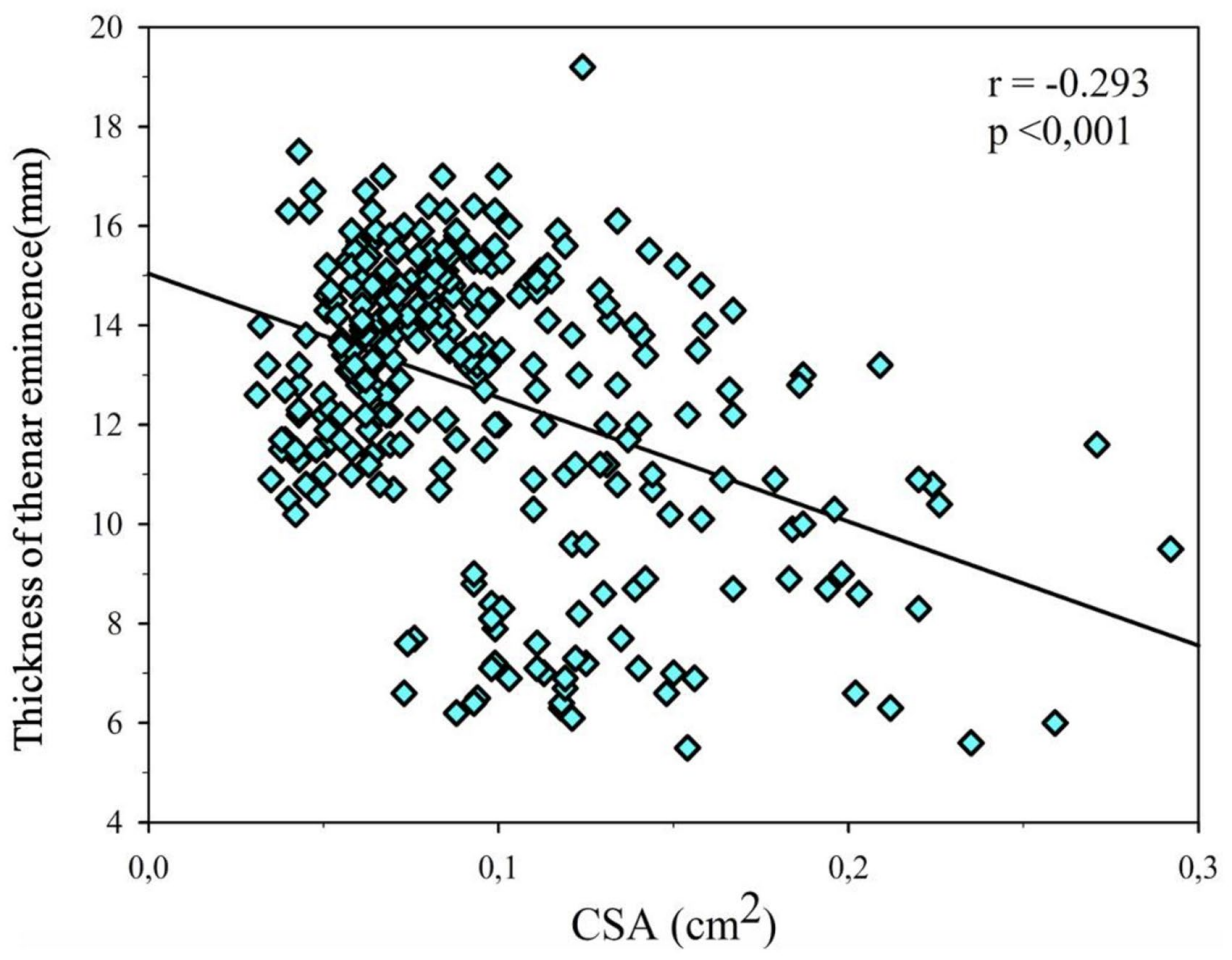
Correlation of median nerve cross-sectional area and thenar muscle thickness.

A statistically significant but weak negative correlation was observed between median nerve CSA and thenar muscle thickness (*r* = −0.293, *p* < 0.05), indicating a limited association between these parameters (Figure 6).

Normality was assessed using the Kolmogorov–Smirnov test. All variables were normally distributed; therefore, data are presented as mean ± standard deviation.

## 4 Discussion

The present study demonstrated that ultrasonography has excellent diagnostic accuracy in the evaluation of carpal tunnel syndrome, as reflected by a high AUC value. Median nerve cross-sectional area increased with disease severity, whereas thenar muscle thickness decreased significantly. Although a statistically significant correlation was identified between these parameters, the strength of this association was weak. Overall, these findings highlight the diagnostic value of ultrasonography in CTS.

The CTS is typically diagnosed based on clinical findings and electrophysiological studies; however, diagnostic challenges may arise in cases with underlying polyneuropathy, prior surgery, or advanced disease stages where nerve conduction responses are absent. In this context, ultrasonography has emerged as a valuable adjunctive modality due to its non-invasive nature, accessibility, and ability to provide anatomical information. Its increasing use in clinical practice reflects its utility, particularly in complex or inconclusive cases (11, 13, 14).

Our findings are consistent with previous and recent studies demonstrating the diagnostic utility of median nerve CSA. Recent literature (2023–2026) has reported high diagnostic accuracy of CSA measurements, with AUC values frequently exceeding 0.90 (9). Furthermore, previously reported cutoff values for CSA vary across studies, generally ranging between 0.08 and 0.11 cm^2^ (10). In the literature, statistical analyses have found critical values for the median nerve CSA of 9 mm^2^ to 15 mm^2^. Sensitivity was determined as 70–88% and specificity as 57–97% (12, 15–19). In our study, the optimal cutoff value of 0.0905 cm^2^, with high sensitivity and specificity, falls within this range and supports the reliability of ultrasonographic assessment. The variability in reported cutoff values may be attributed to differences in study populations, measurement techniques, and diagnostic criteria.

In the present study, the median nerve cross-sectional area (CSA) measured at the carpal tunnel inlet demonstrated high diagnostic accuracy for carpal tunnel syndrome (20). These findings are consistent with those reported by Mohamed et al (26) who also identified the inlet level as the most diagnostically relevant site for CSA measurement. In their study, the mean CSA at the inlet was significantly higher in CTS patients compared to controls (0.14 ± 0.03 cm^2^ vs. 0.09 ± 0.002 cm^2^), and an optimal cut-off value of ≥8.8 mm^2^ was proposed, yielding a sensitivity of 97.1% and specificity of 85.7%.

Similarly, in our study, CSA values at the carpal tunnel inlet were significantly increased in CTS patients, with a comparable diagnostic performance demonstrated by ROC analysis. However, the slight differences in cut-off values between studies may be attributed to variations in study populations, measurement techniques, and ultrasound protocols. These findings further support the importance of standardizing CSA measurement at the carpal tunnel inlet and suggest that population-specific reference values may be necessary for optimal diagnostic accuracy.

In addition to its diagnostic performance, the relationship between median nerve cross-sectional area (CSA) and disease severity has been widely investigated. In a recent study by Kara et al., CSA demonstrated low-to-moderate correlations with electrodiagnostic (EDX) parameters, including distal motor latency and sensory conduction abnormalities, while no significant association was observed with overall clinical symptom burden (21).

Similarly, in our study, CSA measurements were significantly associated with electrophysiological findings, supporting the concept that nerve enlargement reflects underlying structural and functional impairment. However, consistent with the findings of Kara et al., the strength of these associations was limited, suggesting that CSA alone may not fully capture disease severity. These results indicate that while ultrasonographic CSA is a valuable morphological parameter, it should be interpreted in conjunction with electrophysiological and clinical assessments rather than being used as a standalone indicator of CTS severity.

In addition to CSA, thenar muscle thickness was significantly reduced in CTS patients, particularly in advanced stages, indicating muscle atrophy associated with chronic nerve compression. However, no significant differences were observed in hypothenar muscle thickness, suggesting that thenar muscle evaluation may be more relevant in CTS assessment. The weak negative correlation between CSA and thenar thickness further supports that these parameters reflect different aspects of disease pathology (22). The weak negative correlation between CSA and thenar thickness further supports that these parameters reflect different aspects of disease pathology. While CSA primarily reflects nerve swelling due to compression, thenar muscle changes are more closely related to chronic denervation and disease duration, which may explain the limited strength of the observed association (23).

Previous studies have also investigated the role of quantitative muscle ultrasonography in CTS. For example, Lee et al. reported decreased thenar muscle thickness and increased echogenicity in CTS patients, supporting the role of muscle assessment in disease evaluation (14). Our findings are in agreement with these observations, although the strength of the association between CSA and muscle thickness was limited in our cohort.

Ultrasonography also enables the detection of anatomical variations, such as bifid median nerve, which may have clinical and surgical implications (24, 25). In our study, the frequency of bifid median nerve was comparable to previously reported rates. Identification of such variations is important, particularly in patients undergoing surgical intervention.

From a clinical perspective, ultrasonography appears to be a valuable non-invasive tool in the evaluation of CTS. Given its high diagnostic accuracy, it may serve as a useful adjunct to electrophysiological studies, particularly in patients with inconclusive ENMG findings or in those who cannot tolerate electrophysiological testing. However, despite its advantages, ultrasonography should not be considered a standalone screening or replacement modality, and its use is best integrated within a comprehensive diagnostic approach.

The findings of this study should be interpreted in light of certain methodological considerations. Since the unit of analysis was the wrist, the inclusion of bilateral wrists from the same patient may have introduced intra-individual correlation, potentially affecting the independence of observations.

The present study has several limitations. First, this was a single-center study, which may limit the generalizability of the findings. Second, the wrist-based analysis was performed without adjustment for clustering, which may have introduced intra-individual correlation. Third, ultrasonographic examinations were performed by a single operator, which may limit reproducibility. Finally, the selection of the control group may have introduced potential selection bias. These limitations should be considered when interpreting the results.

## 5 Conclusion

Ultrasonography demonstrated high diagnostic accuracy in the evaluation of carpal tunnel syndrome and showed good agreement with electrophysiological findings. Quantitative parameters, particularly median nerve cross-sectional area and thenar muscle thickness, provided valuable diagnostic information. However, ultrasonography should be considered a complementary tool rather than a replacement for electrophysiological studies. Future multicenter studies with larger sample sizes and standardized measurement protocols are needed to further validate these findings and to establish widely accepted diagnostic cutoff values.

## Data Availability

The original contributions presented in the study are included in the article/supplementary material, further inquiries can be directed to the corresponding author.

## Author’s note

This study was presented at the 60th National Neurology Congress, on November 26-December 2, 2024 in Antalya, Turkey.
